# Monitoring the electric activity of the diaphragm during noninvasive positive pressure ventilation: a case report

**DOI:** 10.1186/s12890-017-0434-2

**Published:** 2017-06-17

**Authors:** Fabia Diniz-Silva, Anna Miethke-Morais, Adriano M. Alencar, Henrique T. Moriya, Pedro Caruso, Eduardo L. V. Costa, Juliana C. Ferreira

**Affiliations:** 1 Pulmonary Division, Heart Institute (InCor) – Hospital das Clínicas da Faculdade de Medicina da Universidade de São Paulo, São Paulo, Brazil; 2 Physics Institute, University of São Paulo, São Paulo, Brazil; 3 Biomedical Engineering Laboratory, University of São Paulo, São Paulo, Brazil

**Keywords:** Case reports, Positive-pressure respiration, Respiration, artificial, Ventilator weaning, Noninvasive ventilation, Diaphragm

## Abstract

**Background:**

In patients with post-extubation respiratory distress, delayed reintubation may worsen clinical outcomes. Objective measures of extubation failure at the bedside are lacking, therefore clinical parameters are currently used to guide the need of reintubation. Electrical activity of the diaphragm (EAdi) provides clinicians with valuable, objective information about respiratory drive and could be used to monitor respiratory effort.

**Case presentation:**

We describe the case of a patient with Chronic Obstructive Pulmonary Disease (COPD), from whom we recorded EAdi during four different ventilatory conditions: 1) invasive mechanical ventilation, 2) spontaneous breathing trial (SBT), 3) unassisted spontaneous breathing, and 4) Noninvasive Positive Pressure Ventilation (NPPV). The patient had been intubated due to an exacerbation of COPD, and after four days of mechanical ventilation, she passed the SBT and was extubated. Clinical signs of respiratory distress were present immediately after extubation, and EAdi increased compared to values obtained during mechanical ventilation. As we started NPPV, EAdi decreased substantially, indicating muscle unloading promoted by NPPV, and we used the EAdi signal to monitor respiratory effort during NPPV. Over the next three days, she was on NPPV for most of the time, with short periods of spontaneous breathing. EAdi remained considerably lower during NPPV than during spontaneous breathing, until the third day, when the difference was no longer clinically significant. She was then weaned from NPPV and discharged from the ICU a few days later.

**Conclusion:**

EAdi monitoring during NPPV provides an objective parameter of respiratory drive and respiratory muscle unloading and may be a useful tool to guide post-extubation ventilatory support. Clinical studies with continuous EAdi monitoring are necessary to clarify the meaning of its absolute values and changes over time.

## Background

Managing severe exacerbations of chronic obstructive pulmonary disease (COPD) can be challenging, both during invasive mechanical ventilation (MV) and after extubation, when noninvasive positive pressure ventilation (NPPV) plays an important role [1–3].

The best strategy for monitoring clinical response to NPPV after extubation is not clear. Clinical parameters, oxygen saturation, and blood gas analysis are recommended to guide whether the patient requires reintubation or can be weaned from NPPV [4], but the lack of objective measures of respiratory overload at the bedside may contribute to delayed reintubation and worse clinical outcomes [5, 6].

Monitoring of the electrical activity of the diaphragm (EAdi) can provide objective information about the neural respiratory drive, since its amplitude reflects the neural excitation of the diaphragm. EAdi monitoring has been an important research tool to help us understand respiratory drive activation and neuro-muscular coupling [7].

**Fig. 1:**
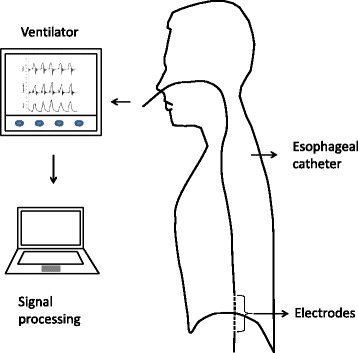
Illustration of the NAVA catheter positioning and data collection apparatus. NAVA: Neurally adjusted ventilatory assist

EAdi can be captured and recorded using an esophageal catheter with multiple electrodes [7, 8] (Fig. 1). It is used to trigger and to provide inspiratory support proportional to the electrical activity of the diaphragm in the ventilatory mode called Neurally adjusted ventilatory Assist (NAVA) [9, 10]. EAdi monitoring has also been used to monitor respiratory effort during the weaning phase in invasive mechanical ventilation [11–13], but not to monitor post-extubation NPPV effectiveness. We present a case of EAdi monitoring during post-extubation NPPV in a patient with an exacerbation of COPD. We followed the CARE guidelines for reporting of case reports [14] and we obtained written approval to publish the case report from the patient.

## Case presentation

A 60-year-old woman with a previous history of severe COPD classified as GOLD D [15] was admitted to the hospital with acute hypercapnic respiratory failure due to a COPD exacerbation. She reported three days of worsened dyspnea, larger production of sputum and low fever. She was an ex-smoker of 120 pack.years and had been diagnosed with COPD 10 years earlier. Despite optimized medical treatment, she had limiting dyspnea and severe obstruction on pulmonary function tests (FEV_1_ of 0.48 L-19% of its predicted value). In the emergency room, she was initially treated with NPPV, but after 30 min her respiratory pattern had not improved, arterial blood gas analysis showed a pH = 7.18 and PCO_2_ = 82, so she was intubated, mechanically ventilated and transferred to the intensive care unit (ICU).

**Fig. 2:**
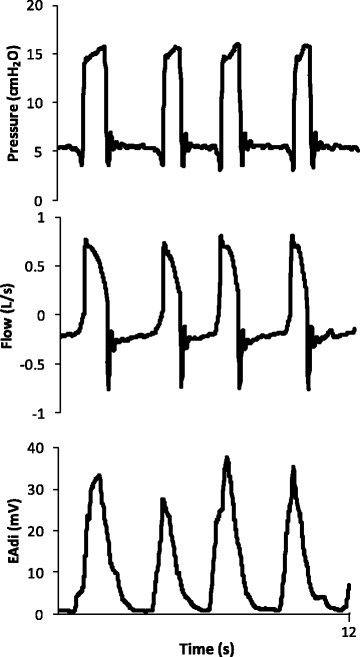
Electrical activity of the diaphragm (EAdi), flow and airway pressure (Paw) during invasive mechanical ventilation in pressure support of 10 cmH_2_O, with PEEP of 5 cmH_2_O. Captured at 100Hz and processed with MatLab

On the first two days of mechanical ventilation she was sedated with fentanyl and propofol and ventilated in pressure controlled mode using an ICU ventilator (Servoi, Maquet, Sweden). She received antibiotics, bronchodilators, and methylprednisolone, and on the third, day sedation was changed to dexmedetomedyne and the ventilatory mode was changed to pressure support, with a PEEP of 5 cmH_2_O, pressure support of 10 cmH_2_O over PEEP, and FIO_2_ of 50%. On the fourth day on mechanical ventilation she showed significant improvement and dexmedetomedyne was reduced to low doses to submit her to a spontaneous breathing trial (SBT). An EAdi catheter (Maquet, Sweden) was inserted as part of a clinical trial (NCT01137271), which evaluated Neurally Adjusted Ventilatory Assist (NAVA) during spontaneous breathing trials (SBT). The catheter was positioned according to manufacturer’s instructions and adequate position was checked using a specific screen in the ventilator, similarly to previous studies [16] and its adequate position was checked before each recording. EAdi was captured at 100Hz using ServoTracker v4.0 (Maquet, Sweden) for five minutes at each situation of interest. We processed and analyzed the data using MatLab (Mathworks, MA, USA), which automatically detected the initiation and termination of inspiratory efforts, and calculated peak EAdi. Respiratory rate (RR) was calculated by dividing the number of respiratory cycles by the number of minutes in each recording. We excluded cycles with artifacts and averaged all the cycles in each five-minute recording to generate mean values for EAdi for each situation [17] (Fig. 2).

**Fig. 3:**
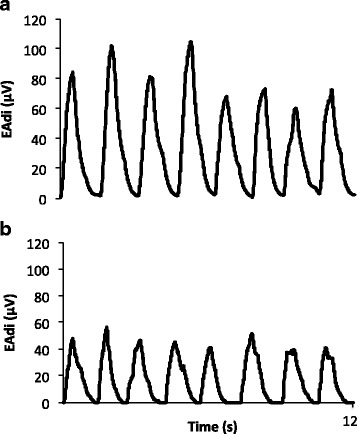
EAdi during SB (**a**) and NPPV (**b**) shown for 12 s on day 1. During NPPV, EAdi decreases and respiratory rate is lower compared to SB. NPPV: Noninvasive Positive Pressure Ventilation; SB: Spontaneous breathing; EAdi: Electrical activity of the diaphragm

The patient was submitted to an SBT in pressure support, with PEEP of 5 cmH_2_O and pressure support of 5 cmH_2_O, for 30 min. We used standard criteria of SBT failure, such as respiratory rate greater than 35, reduced tidal volume, agitation or diaphoresis, hypoxemia, among others. She didn’t present any of these criteria and was extubated. In our ICU, we regularly use NPPV after extubation to prevent acute respiratory failure for high risk patients, and a few minutes after extubation, while we were preparing to install NPPV, we observed clinical signs of increased respiratory effort, including retraction of the intercostal spaces and accessory muscle recruitment. At this moment, we noted that EAdi was higher than during MV, over 90 μV. After the start of NPPV, EAdi decreased substantially, which was compatible with muscle unloading promoted by NPPV (Fig. 3). Over the following days, the EAdi signal was used to monitor and compare respiratory effort during NPPV and spontaneous breathing (Table 1).

**Table 1:** Respiratory parameters from the last day of invasive mechanical ventilation to the third day after extubation

Variable	PSV	SBT	Post extubation NPPV	Day 1		Day 2		Day 3	
				NPPV	SB	NPPV	SB	NPPV	SB
EAdi (μV)	23.3	23.9	63.0	45.1	76.4	39.4	83.7	59.2	64.9
RR (bpm)	21	24	24	22	24	27	21	27	28
Expiratory pressure (cmH_2_O)	5	5	5	5	–	8	–	8	–
Inspiratory pressure (cmH_2_O)	15	10	15	12	–	10	–	10	–

*PSV* Pressure Support Ventilation, *SBT* Spontaneous breathing trial, *NPPV* Noninvasive Positive Pressure Ventilation, *SB* Spontaneous breathing, *EAdi* Electrical activity of the diaphragm, in microvolts (μV), *RR* Respiratory Rate in breaths per minute, *Inspiratory pressure* peak airway pressure during inspiration

The patient was kept in NPPV in a dedicated NPPV ventilator (V60, Philips, The Netherlands) in bi-level mode (inspiratory support over an expiratory pressurization), with a total face mask (Performax, Phillips Respironics, PA, USA) almost continuously for the next two days, with 30-min intervals of spontaneous breathing with a nasal oxygen catheter every six hours. On NPPV, the patient reported less dyspnea, and clinical signs of respiratory effort were less prominent as compared to spontaneous breathing. She received clonazepam 2 mg twice a day and quetiapine 50 mg twice a day to control anxiety and facilitate sleep.

**Fig. 4:**
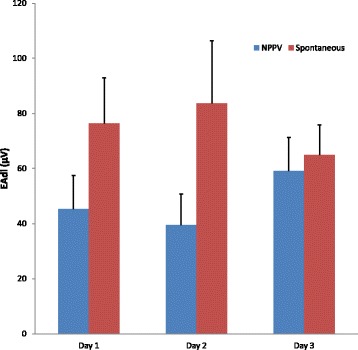
Mean EAdi values during NPPV (*blue bars*) and spontaneous breathing (*red bars*). Notice that EAdi is smaller on NPPV compared to spontaneous breathing on Days 1 and 2, but not on Day 3. Bars represent the mean and error bars represent the standard deviation. EAdi: Electrical activity of the diaphragm; NPPV: Noninvasive Positive Pressure Ventilation

Once a day, we recorded EAdi for the last 5 min of NPPV and for the last 5 min of spontaneous breathing. On the first two days, EAdi gradually decreased on NPPV (Table 1, Fig. 4).

On the third day, EAdi on NPPV was no longer significantly lower than in spontaneous breathing (Table 1) and NPPV was weaned. After 6 days of ICU stay, she was discharged to a clinical ward, and three days later she was discharged home.

## Discussion

We describe the use of EAdi to monitor the response to post-extubation NPPV in a patient with severe COPD and high risk of reintubation. EAdi monitoring provided an objective measure of the respiratory muscle unloading promoted by post-extubation NPPV, which encouraged us to maintain NPPV, when clinical signs could have led us to reintubation.

The observation that NPPV led to a significant reduction of EAdi compared to spontaneous breathing suggested that NPPV was indeed providing significant muscle unloading, and this information was important for our decision to keep NPPV instead of early reintubation. Without the EAdi information, we would need to rely solely on clinical signs of intolerance, such as accessory muscle use and increased respiratory rate, which can be very subjective, especially in patients with chronic lung diseases such as COPD. While absolute values of EAdi were quite high in both NPPV and spontaneous breathing, the decrease of EAdi with NPPV compared to spontaneous breathing was an objective measure of muscle unloading.

Monitoring of EAdi has recently been proposed as a predictor of weaning failure. Barwing and colleagues monitored EAdi during SBTs and found that patients who failed their SBT had a more pronounced increase in EAdi than patients who passed the SBT. This increase was noticeable earlier than changes in conventional clinical parameters [11]. Dres and colleagues monitored EAdi in 57 patients during SBTs and found that while EAdi and EAdi-derived indices, such as EAdi normalized by the tidal volume, were good predictors of weaning failure, they were not superior to the rapid shallow breathing index. Interestingly, they found that EAdi was significantly higher at baseline in patients who failed the weaning process [12]. Liu and colleagues studied the neuroventilatory efficiency (NVE) during SBTs, i.e., the ability to generate tidal volume normalized by the neural drive, calculated as the tidal volume divided by EAdi. They found that NVE is a good predictor of weaning failure, and is superior to the rapid shallow breathing index [13].

For extubated patients, EAdi changes over time have been described in a case report in a patient with post-extubation respiratory failure treated with noninvasive ventilation using NAVA. In that case, EAdi decreased abruptly after two hours of NPPV, causing a reduction in tidal volume and leading to reintubation. The authors hypothesized that the abrupt fall in EAdi might have been a consequence of low-frequency fatigue of the diaphragm [18].

EAdi values during NPPV have also been reported in studies comparing noninvasive NAVA and noninvasive pressure support, but no cut-off values are available [19, 20]. Interestingly, some patients in these studies had of high values of EAdi but none of them compares EAdi during NPPV with spontaneous breathing. EAdi monitoring after extubation is a new tool and a few aspects of its clinical interpretation were particularly challenging in our patient. EAdi values after extubation were substantially high and could have been interpreted as a sign of need for reintubation. However, EAdi has no established normal cut-off values, previous studies report a large inter subject variability and similarly high values have been reported during the SBT in successfully weaned patients [8, 11, 12]. Our clinical interpretation of the data was that lower EAdi with NPPV when compared to SB was more important than absolute EAdi values. On the third day, EAdi on NPPV was no longer different from spontaneous breathing, because, compared to the previous day, EAdi increased during NPPV and decreased during unassisted breathing.. The increase in EAdi during NPPV might have been related to intra subject variability of EAdi [21], decreased tolerance of the facial mask or subclinical agitation, and emphasizes the importance of combining objective EAdi measures with clinical assessment. Since the patient was clinically stable, we extended the duration of spontaneous breathing and rapidly weaned NPPV.

To our knowledge, there are no reports of EAdi monitoring during NPPV and spontaneous breathing after extubation. Our data showed that the contrast between EAdi on NPPV with EAdi during spontaneous breathing was helpful to guide NPPV application in this case. This report offers an insight into the potential utility of EAdi during the application of post-extubation NPPV, but needs further studying.

There are some limitations to our report that should be acknowledged. First, we report just one case, and individual particularities prevent extrapolations to other patients. Second, tidal volume recording was available only while the patient was intubated, since our recoding system is not compatible with the NPPV ventilator. Finally, the lack of normal cut off values for EAdi and its considerable variability documented in previous studies makes the interpretation of the high absolute values we report difficult. Using EAdi changes over time may prove useful but more studies are needed to help us better understand the role of EAdi monitoring during the weaning and post extubation phases.

## Conclusions

EAdi monitoring during NPPV provides an objective parameter of respiratory drive and respiratory muscle unloading and may be a useful tool to guide mechanical ventilation weaning and post-extubation ventilatory support. Clinical studies with continuous EAdi monitoring are necessary to clarify the meaning of its absolute values and changes over time.

## Abbreviations

CARE: CAse REports
COPD: Chronic obstructive pulmonary disease
EAdi: Electrical activity of the diaphragm
EPAP: Expiratory positive airway pressure
FEV_1_: Forced expiratory volume on the first second
ICU: Intensive care unit
IPAP: Inspiratory positive airway pressure
MV: Mechanical ventilation
NAVA: Neurally adjusted ventilatory assist
NPPV: Noninvasive positive pressure ventilation
NVE: Neuroventilatory efficiency
Paw: Peak airway pressure
PEEP: Positive end-expiratory pressure
PSV: Pressure support ventilation
RR: Respiratory rate
SB: Spontaneous breathing
SBT: Spontaneous breathing trial

## References

[1] Boles J-M, Bion J, Connors A, Herridge M, Marsh B, Melot C (2007). Weaning from mechanical ventilation. Eur Respir J [Internet].

[2] Mechanical Ventilation Committee of the Brazilian Intensive Care Medicine Association, Commission of Intensive Therapy of the Brazilian Thoracic Society (2014). Brazilian recommendations of mechanical ventilation 2013. Part I. J Bras Pneumol.

[3] Dres M, Tran T-C, Aegerter P, Rabbat A, Guidet B, Huchon G (2013). Influence of ICU case-volume on the management and hospital outcomes of acute exacerbations of chronic obstructive pulmonary disease*. Crit Care Med [Internet].

[4] BTS Guideline. Non-invasive ventilation in acute respiratory failure. Thorax [Internet]. 2002;57:192–211. Available from: http://dx.doi.org/10.1136/thorax.57.3.192. [cited 2013 Jun 26]. 10.1136/thorax.57.3.192 PMC1746282 11867822

[5] Esteban A, Frutos-Vivar F, Ferguson ND, Arabi Y, Apezteguía C, González M, et al.Noninvasive positivepressure ventilation for respiratory failure after extubation. N Engl J Med [Internet]. 2004;350:2452–60. Available from: http://www.nejm.org/doi/full/10.1056/NEJMoa032736. 10.1056/NEJMoa032736 15190137

[6] Ferreira JC, Medeiros P, Rego FM, Caruso P. Risk factors for noninvasive ventilation failure in cancer patients in the intensive care unit: A retrospective cohort study. J Crit Care [Internet]. 2015;30:1003–7. Available from: http://www.sciencedirect.com/science/article/pii/S0883944115002683. [cited 2015 Sep 2]. 10.1016/j.jcrc.2015.04.121 26072388

[7] Beck J, Gottfried SB, Navalesi P, Skrobik Y, Comtois N, Rossini M, et al. Electrical activity of the diaphragm during pressure support ventilation in acute respiratory failure. Am J Respir Crit Care Med [Internet. 2001;164:419–24. Available from: https://doi.org/10.1164/ajrccm.164.3.2009018. 10.1164/ajrccm.164.3.2009018 11500343

[8] Doorduin J, van Hees HWH, van der Hoeven JG, Heunks LMA. Monitoring of the respiratory muscles in the critically ill. Am J Respir Crit Care Med [Internet]. 2013;187:20–7. Available from: https://doi.org/10.1164/rccm.201206-1117CP. 10.1164/rccm.201206-1117CP 23103733

[9] Sinderby C, Navalesi P, Beck J, Skrobik Y, Comtois N, Friberg S, et al. Neural control of mechanical ventilation in respiratory failure. Nat Med [Internet]. 1999;5:1433–6. Available from: https://www.nature.com/nm/journal/v5/n12/full/nm1299_1433.html. 10.1038/71012 10581089

[10] Terzi N, Piquilloud L, Rozé H, Mercat A, Lofaso F, Delisle S, et al. Clinical review: Update on neurally adjusted ventilatory assist - report of a round-table conference. Crit Care [Internet]. 2012;16:225. Available from: https://ccforum.biomedcentral.com/articles/10.1186/cc11297. 10.1186/cc11297 PMC3580602 22715815

[11] Barwing J, Pedroni C, Olgemöller U, Quintel M, Moerer O. Electrical activity of the diaphragm (EAdi) as a monitoring parameter in difficult weaning from respirator: a pilot study. Crit Care [Internet]. 2013;17:R182. Available from: https://doi.org/10.1186/cc12865. [cited 2014 Sep 24]. 10.1186/cc12865 PMC4057029 23985299

[12] Dres M, Schmidt M, Ferre A, Mayaux J, Similowski T, Demoule A. Diaphragm electromyographic activity as a predictor of weaning failure. Intensive Care Med. 2012;38:2017–25. Available from: https://doi.org/10.1007/s00134-012-2700-3. 10.1007/s00134-012-2700-3 23011532

[13] Liu L, Liu H, Yang Y, Huang Y, Liu S, Beck J, et al. Neuroventilatory efficiency and extubation readiness in critically ill patients. Crit Care [Internet]. 2012;16:R143. BioMed Central Ltd, [cited 2014 Oct 6]. Available from: https://doi.org/10.1186/cc11451. 10.1186/cc11451 PMC3580730 22849707

[14] Gagnier JJ, Kienle G, Altman DG, Moher D, Sox H, Riley D (2013). The CARE guidelines: consensus-based clinical case reporting guideline development. Case Reports [Internet].

[15] From the Global Strategy for the Diagnosis, Management and Prevention of COPD, Global Initiative for Chronic Obstructive Lung Disease (GOLD). 2016 [Internet]. Available from: http://goldcopd.org/global-strategydiagnosis-management-prevention-copd-2016/.

[16] Barwing J, Ambold M, Linden N, Quintel M, Moerer O. Evaluation of the catheter positioning for neurally adjusted ventilatory assist. Intensive Care Med [Internet]. 2009;35:1809–14. Available from: https://doi.org/10.1007/s00134-009-1587-0. [cited 2013 Jun 25]. 10.1007/s00134-009-1587-0 PMC2749172 19652950

[17] Ferreira JC, Diniz-Silva F, Moriya HT, Alencar AM, Amato MBP, Carvalho CRR. Patient-Ventilator Interaction In Neurally Adjusted Ventilatory Assist (NAVA) And Pressure Support (PSV) During Spontaneous Breathing Trials. C45. Mech. Vent. [Internet]. American Thoracic Society; 2014. p. A4571–A4571. Available from: http://www.atsjournals.org/doi/abs/10.1164/ajrccm-conference.2014.189.1_MeetingAbstracts.A4571.

[18] Rozé H, Richard JCM, Mercat A, Brochard L. Recording of possible diaphragm fatigue under neurally adjusted ventilatory assist. Am J Respir Crit Care Med [Internet]. 2011;184:1213–4. Available from: http://www.ncbi.nlm.nih.gov/pubmed/22086994. 10.1164/ajrccm.184.10.1213a 22086994

[19] Doorduin J, Sinderby CA, Beck J, van der Hoeven JG, Heunks LMA. Automated patient-ventilator interaction analysis during neurally adjusted non-invasive ventilation and pressure support ventilation in chronic obstructive pulmonary disease. Crit Care [Internet]. 2014;18:550. Available from: https://ccforum.biomedcentral.com/articles/10.1186/s13054-014-0550-9. 10.1186/s13054-014-0550-9 PMC4207887 25307894

[20] Schmidt M, Dres M, Raux M, Deslandes-Boutmy E, Kindler F, Mayaux J, et al. Neurally adjusted ventilatory assist improves patient-ventilator interaction during postextubation prophylactic noninvasive ventilation. Crit Care Med. 2012;40:1738–44. Available from: https://www.ncbi.nlm.nih.gov/pubmed/22610179. 10.1097/CCM.0b013e3182451f77 22610179

[21] Tobin MJ. Probing with the ventilator. Crit Care [Internet]. 2013;17:198. Available from: https://ccforum.biomedcentral.com/articles/10.1186/cc13038. 10.1186/cc13038 PMC4056809 24090345

